# Nucleotide excision repair in tumor response to DNA-damaging anticancer therapies: a comprehensive review

**DOI:** 10.1007/s00432-026-06563-0

**Published:** 2026-07-22

**Authors:** Bartosz Maćkowiak, Kamila Ostrowska, Julia Kozikowska, Katarzyna Kulcenty, Wojciech Golusiński, Wiktoria Suchorska

**Affiliations:** 1 Radiobiology Laboratory, Greater Poland Cancer Centre, Garbary 15 Street, Poznan, 61-866 Poland; 2 Doctoral School of Molecular Medicine, Medical University of Lodz, Haller Square 1, Lodz, 90-624 Poland; 3 Department of Clinical Chemistry and Molecular Diagnostics, Poznan University of Medical Sciences, Rokietnicka 3 Street, Poznan, 60-806 Poland; 4 Department of Head and Neck Surgery, Poznan University of Medical Sciences, Garbary 15 Street, Poznan, 61-866 Poland; 5 Department of Electroradiology, Poznan University of Medical Sciences, Fredry 10 Street, Poznan, 61-701 Poland

**Keywords:** DNA damage repair, Nucleotide excision repair, Cancer therapy, Biomarkers

## Abstract

Nucleotide excision repair (NER) is a critical DNA repair pathway responsible for removing bulky, helix-distorting DNA lesions, including ultraviolet radiation-induced photoproducts and platinum drug-DNA adducts. This review provides a comprehensive overview of current understanding of NER in cancer, with particular emphasis on its role in therapeutic response. We discuss the molecular mechanisms of DNA repair in cancer, the significance of NER in platinum-based therapies, results from in vitro studies across various cancer types, findings from patient-based studies, and outcomes from clinical trials. Special attention is given to ERCC1, ERCC2/XPD, XPA, and XPC molecules as potential biomarkers of treatment response and therapeutic targets, and incorporating the most recent discoveries regarding NER inhibitors, mutational signatures, and synthetic lethality-based approaches.

**Graphical abstract:**

**Figure:**
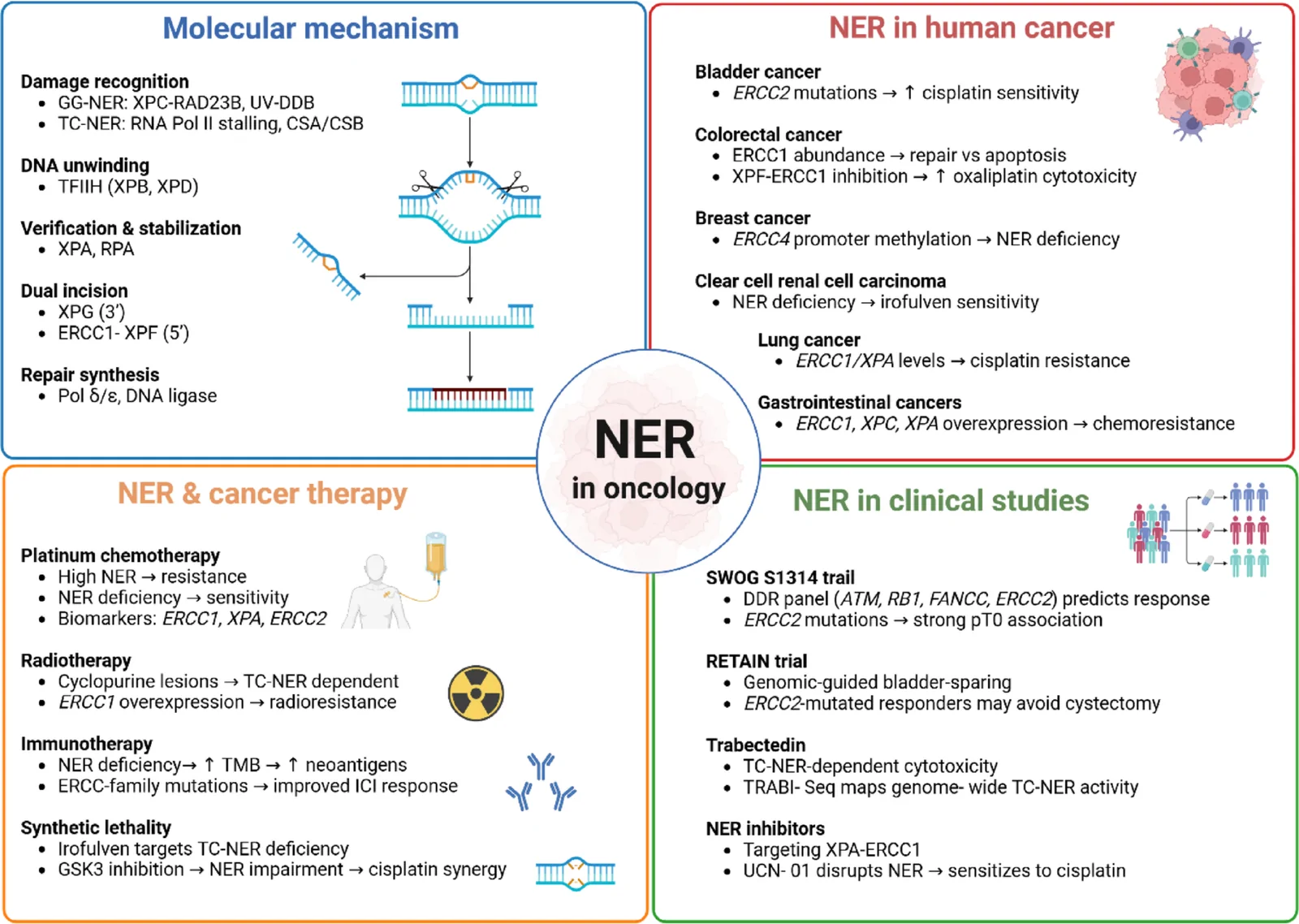

## DNA repair mechanisms in cancer

Genomic instability is one of the fundamental hallmarks of malignant neoplasms, enabling the accumulation of mutations and disease progression (Hanahan 2022). Human cells possess a complex DNA repair system with several distinct pathways, each specializing in the removal of specific types of DNA damage (Huang and Zhou 2021; Li et al. 2023). The major DNA repair pathways include: base excision repair (BER), mismatch repair (MMR), nucleotide excision repair (NER), homologous recombination (HR), and non-homologous end joining (NHEJ) (Huang and Zhou 2021).

**Fig. 1:**
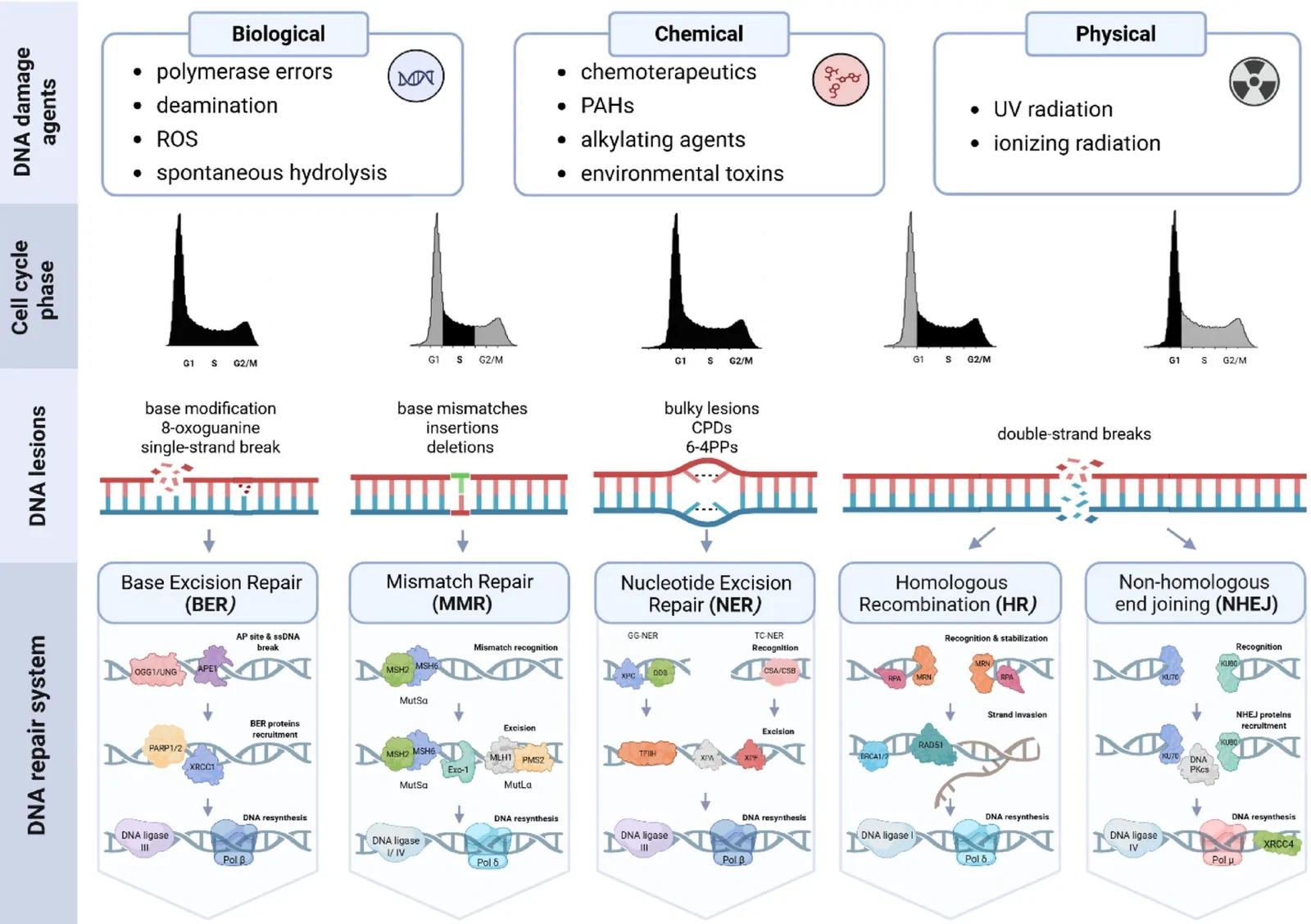
Major causes of DNA damage and cellular repair pathways. Biological, chemical, and physical agents generate distinct types of DNA lesions, including base modifications, mismatches, bulky DNA adducts, and double-strand breaks. These lesions are repaired in selected cell cycle phases (black) by specialized pathways including Base Excision Repair (BER), Mismatch Repair (MMR), Nucleotide Excision Repair (NER), Homologous Recombination (HR), and Non-homologous End Joining (NHEJ). Together, these systems maintain genomic stability and prevent mutagenesis.

In cancer cells, the balance between DNA damage and repair is frequently disrupted. On the one hand, defects in DNA repair genes predispose to cancer development by accumulating mutations. On the other hand, the same repair pathways can protect cancer cells from the effects of DNA-damaging anticancer agents, leading to chemotherapy resistance (Reed 1998; Kelland 2007). Understanding this dual role of DNA repair is essential for developing effective therapeutic strategies. Figure 1 summarizes the molecular mechanisms of major DNA repair pathways.

### Overview of major DNA repair pathways

DNA repair pathways coordinate interconnected mechanisms to preserve genomic stability, modulate cellular responses to endogenous and therapy-induced DNA damage, and significantly influence cancer development. (Torgovnick and Schumacher 2015), treatment sensitivity and resistance (Li et al. 2021), and post-treatment regeneration processes (Chen et al. 2024). To date, several DNA repair pathways have been identified, including base excision repair, mismatch repair, homologous recombination, non-homologous end joining, and nucleotide excision repair. Among these mechanisms, this review emphasizes nucleotide excision repair, as this pathway has emerged as a potentially important yet underexplored determinant of cancer biology and response to anticancer therapy.

#### Base excision repair (BER)

The BER pathway is responsible for removing small base modifications such as oxidative damage (e.g., 8-oxoguanine) and base deamination. BER primarily repairs non-bulky lesions that do not cause distortion of the DNA helix, in contrast to NER, which removes bulky, helix-distorting lesions (Yang 2008; Lee and Kang 2019). The process is initiated by DNA glycosylases that recognize and remove the damaged base, creating an apurinic/apyrimidinic (AP) site. Subsequently, AP endonuclease 1 (APE1) introduces a single-strand break, and further repair proceeds through the single-strand break repair (SSBR) pathway involving DNA polymerase β and DNA ligase (Krokan and Bjoras 2013; Huang and Zhou 2021). An efficient BER system preserves genomic stability by preventing the accumulation of DNA lesions. In contrast, dysfunction promotes genomic instability and may influence sensitivity to DNA-damaging therapies. (Gohil et al. 2023)

#### Mismatch repair (MMR)

The MMR system corrects replication errors, including single-base mismatches and small insertions and deletions. Key proteins include MSH2 (MutS Homolog 2), MSH6 (MutS Homolog 6), MLH1 (MutL Homolog 1), and PMS2 (Postmeiotic Segregation Increased 2), forming the MutSα and MutLα heterodimers. Defects in MMR genes are characteristic of Lynch syndrome and lead to microsatellite instability (MSI), which has significant therapeutic implications, particularly in the context of immunotherapy (Baretti and Le 2018).

#### Homologous recombination (HR) and non-homologous end joining (NHEJ)

DNA double-strand breaks (DSBs) are repaired primarily by two mechanisms: the high-fidelity homologous recombination (HR) pathway involving *BRCA1*, *BRCA2*, and *RAD51*, and the error-prone non-homologous end joining (NHEJ). HR deficiency (HRD) in tumors with *BRCA1/2* mutations underlies the efficacy of PARP inhibitors, representing a paradigm for exploiting synthetic lethality in oncology (Farmer et al. 2005; Lord and Ashworth 2017). The clinical success of PARP inhibitors has inspired the search for analogous strategies targeting other DNA repair pathways, including NER.

#### Nucleotide excision repair (NER)

Nucleotide excision repair (NER) is a highly conserved mammalian mechanism that removes bulky, helix-destabilizing lesions from DNA to maintain genome stability during replication and translation (Saha et al. 2020). NER identifies and removes ultraviolet (UV) radiation damage, including two main DNA photoproducts: cyclobutane–pyrimidine dimers (CPDs) and 6–4 pyrimidine–primidone photoproducts (6–4PPs) (Zachayus et al. 2025) , as well as chemical DNA adducts like polycyclic aromatic hydrocarbons (PAHs) related to tobacco smoke, cisplatin intrastrand crosslinks, and ROS-generated cyclopurines (Marteijn et al. 2014; Zachayus et al. 2025). If not removed prior to DNA synthesis and bypassed by error-prone translesion DNA polymerases, such lesions give rise to mutations and chromosomal aberrations that are linked to cell death, aging, senescence, increased mutagenesis, and, furthermore, cancer (Marteijn et al. 2014).

### Nucleotide excision repair (NER)—molecular mechanism

**Fig. 2:**
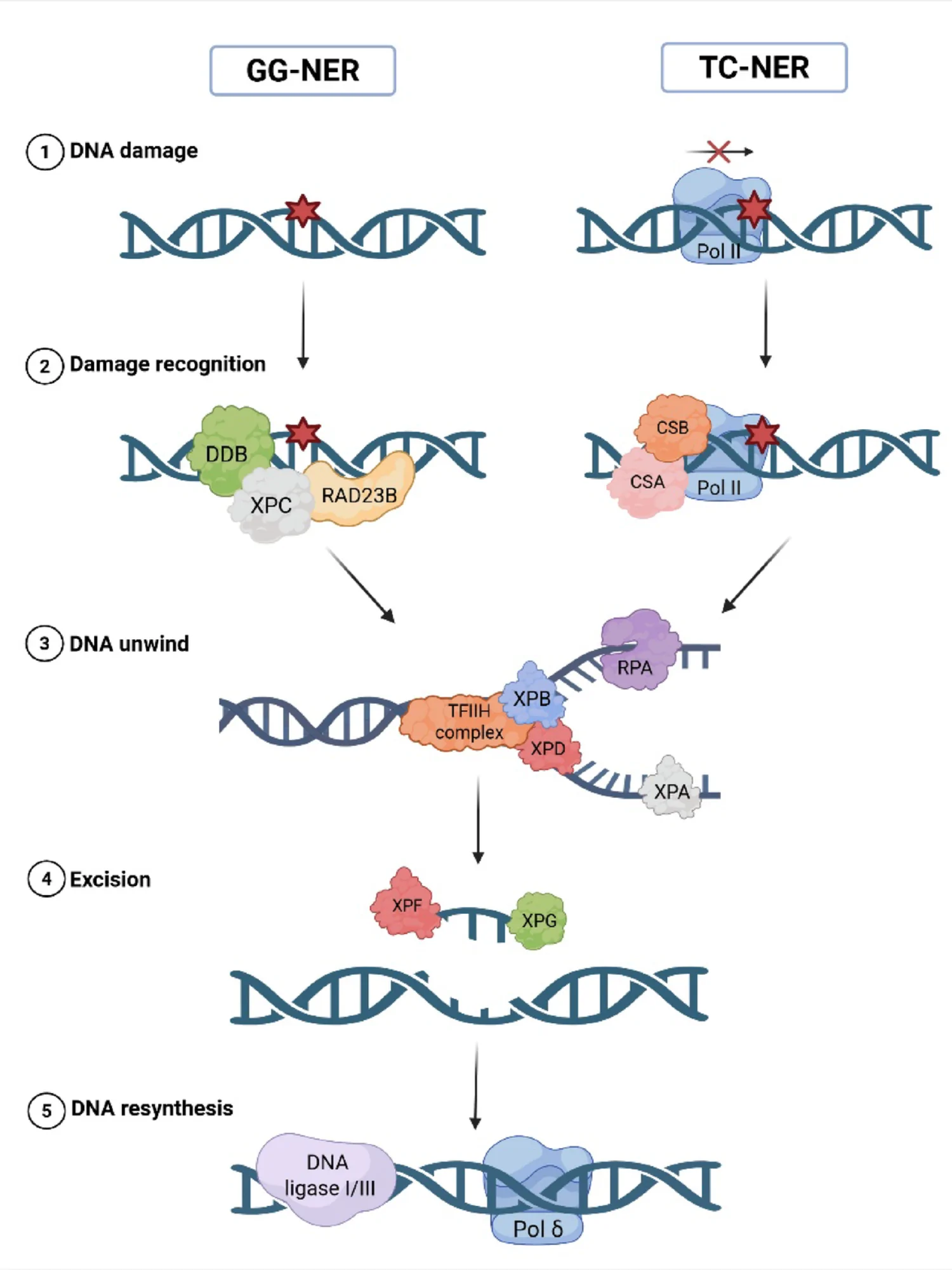
Mechanism of nucleotide excision repair (NER) sub-pathways: global genome NER (GG-NER) and transcription-coupled NER (TC-NER). In GG-NER, DNA lesions are recognized by the DDB and XPC-RAD23B complexes, whereas in TC-NER, stalled RNA polymerase II recruits CSA and CSB proteins. Both pathways recruit the TFIIH (XPB-XPD) complex and, together with RPA and XPA, mediate DNA unwinding and stabilization of single-stranded DNA. Subsequently, the endonucleases XPF and XPG perform a dual incision, followed by DNA resynthesis and ligation, restoring DNA integrity.

In oncology, NER is relevant because it influences genomic stability, cancer susceptibility, and tumor response to DNA-damaging therapies, especially platinum-based compounds (Song et al. 2017). A detailed understanding of the unique molecular pathway of NER is essential to link the mechanistic basis with interpreting tumor sensitivity, adaptive resistance, and biomarker potential in response to DNA-damaging anticancer therapies (Scharer 2013). NER system’s initial recognition can be done in two separate ways: global-genome (GG) or transcription-coupled (TC), as summarized in Fig. 2.

#### Global genome NER (GG-NER)

The GG-NER sub-pathway is responsible for recognizing and repairing lesions throughout the entire genome, regardless of transcriptional activity. Damage recognition is initiated by the XPC-RAD23B complex, which detects helix distortion by binding to the strand opposite the lesion (Sugasawa et al. 1998; Scharer 2013). For certain lesions, such as CPDs, which cause minimal helix distortion, the UV-DDB complex (DDB1-DDB2/XPE) plays an additional role in damage recognition (Scrima et al. 2008). Defects in GG-NER lead to cancer predisposition, as exemplified by xeroderma pigmentosum- an inherited disorder with a thousand-fold increased risk of skin cancer (Friedberg 2001).

#### Transcription-coupled NER (TC-NER)

The TC-NER sub-pathway removes lesions that block RNA polymerase II in actively transcribed genes. The initiating signal is the stalling of elongating RNA polymerase II at the damage site, which leads to the recruitment of CSA (ERCC8) and CSB (ERCC6) proteins (Fousteri and Mullenders 2008; Van Der Weegen et al. 2020). Defects in TC-NER cause Cockayne syndrome, characterized by premature aging but - interestingly - not increased cancer risk (Friedberg 2001). Trabectedin, a drug used in the treatment of sarcomas and ovarian cancer, exploits the TC-NER mechanism in a unique way - it is more toxic to cells with active TC-NER because abortive repair of trabectedin-DNA adducts leads to persistent single-strand breaks (SSBs) (Son et al. 2024).

#### NER molecular mechanisms’ mutual pathway

Following damage recognition by either GG-NER or TC-NER, further repair proceeds through a common pathway. The TFIIH complex, containing the XPB (*ERCC3*) and XPD (*ERCC2*) helicases, unwinds the DNA double helix around the damage site (Scharer 2013). XPA verifies the presence of the lesion and coordinates assembly of the repair complex, while RPA (replication protein A) stabilizes the single-stranded DNA. Subsequently, two endonucleases perform incisions: XPG (*ERCC5*) on the 3’ side of the lesion and the ERCC1-XPF (*ERCC4*) heterodimer on the 5’ side. The excised oligonucleotide fragment is removed, and the resulting gap is filled by DNA polymerase δ/ε and sealed by DNA ligase (De Laat et al. 1999; Scharer 2013; Marteijn et al. 2014). More specifically, repair synthesis may also involve DNA polymerase κ, in addition to DNA polymerases δ and ε (Ogi et al. 2010; Kemp and Hu 2017). Final ligation is mediated by DNA ligase I or by the XRCC1–DNA ligase III complex, with ligase usage influenced by cellular context and proliferative status (Kemp and Hu 2017; Paul-Konietzko et al. 2015).

Among the numerous proteins involved in NER, several have gained particular significance in oncology. ERCC1, which forms a heterodimer with XPF, is responsible for the 5’ incision and serves as a key determinant of overall pathway efficiency. Overexpression of *ERCC1* in cancer cells correlates with cisplatin resistance (Altaha et al. 2004; Olaussen and Postel-Vinay 2016). ERCC2/XPD, as the TFIIH helicase, serves a dual function in repair and transcription, and somatic mutations in ERCC2/XPD are present in approximately 10% of bladder tumors (Van Allen et al. 2014; Börcsök et al. 2025a). XPA serves as a verification and coordination role, while XPC initiates damage recognition in GG-NER. The XPA-ERCC1 interaction is considered one of the most promising pharmacological targets within the NER pathway (Gentile et al. 2016, 2020).

## Dualistic role of NER in cancer treatment

### NER and platinum drug resistance

Platinum drugs (cisplatin, carboplatin, oxaliplatin) are among the most widely used chemotherapeutic agents in oncology. Their mechanism of action involves the formation of interstrand DNA crosslinks that block replication and transcription, leading to apoptosis of cancer cells (Kelland 2007; Li et al. 2023). However, platinum compounds also frequently induce intrastrand DNA crosslinks, especially 1,2-d(GpG), 1,2-d(ApG), and 1,3-d(GpNpG) adducts (Jamieson and Lippard 1999; Wang and Lippard 2005; Duan et al. 2020). These intrastrand lesions represent the major fraction of cisplatin-induced DNA damage and are important substrates for NER (Jamieson and Lippard 1999; Duan et al. 2020). The predominant form of damage consists of 1,2-d(GpG) adducts, which account for approximately 65% of all cisplatin-induced lesions and are removed primarily by the NER pathway (Kelland 2007; Bowden 2014).

The relationship between NER activity and cisplatin sensitivity is inverse: high NER repair capacity leads to efficient removal of platinum-DNA adducts, reducing drug cytotoxicity, whereas diminished NER activity results in increased damage accumulation and greater sensitivity (Welsh et al. 2004; Usanova et al. 2010; Bowden 2014).

Tumor-associated alterations in *XPA* impair NER function, leading to reduced removal of cisplatin-induced DNA adducts and increased cellular sensitivity to the drug. XPA is essential for the proper assembly of the NER pre-incision complex and for the accurate positioning of the *ERCC1*–XPF endonuclease, thereby increasing excision of cisplatin adducts and lowering cytotoxicity (Pajuelo-Lozano et al. 2018). Low *XPA* expression levels are linked to high cisplatin sensitivity in testicular tumors (Zhan et al. 2025). Lower expression of *ERCC1* has been associated with longer median progression-free survival (PFS) and overall survival (OS) in patients with small-cell lung cancer treated with platinum-based chemotherapy (Zou et al. 2023). High *ERCC1*/*XPF* expression levels increase incision efficiency, leading to the elimination of cisplatin adducts before they affect the cell. NER’s signaling network can be co-targeted. Experimental data suggest that modulation of TGF-β signaling alters the expression of multiple NER genes, including *XPA*, *XPB*, and *XPC*, thereby influencing cisplatin resistance (Manguinhas et al. 2024a). Missense mutations in the ERCC2 gene occur in 10% of bladder tumors. They may predict patients’ sensitivity to cisplatin treatment (Börcsök et al. 2025a).

### NER inhibitors as a sensitization strategy

The concept of pharmacologically inhibiting NER to enhance the efficacy of platinum chemotherapy is the subject of intensive research. This strategy is based on the observation that reduced repair of platinum-induced DNA lesions may increase tumor cell sensitivity to platinum compounds (Jiang and Yang 1999; Gentile et al. 2016). Several NER-targeting compounds have been evaluated in preclinical studies, mainly as chemosensitizers rather than as single independent agents (Neher et al. 2010; Jordheim et al. 2013; Gentile et al. 2016). The XPA-ERCC1 interaction represents a particularly attractive target, as it is essential for proper NER function, and *ERCC1* is overexpressed in many tumors (Gentile et al. 2016, 2020). In recent years, several potential inhibitors of this interaction have been identified through virtual screening and in silico drug design methods (Gentile et al. 2016). These compounds are intended to impair recruitment of the ERCC1-XPF complex to the sites of DNA damage, thereby reducing repair efficiency (Gentile et al. 2016, 2020). Additionally, inhibition of GSK3 kinase can functionally impair NER, thereby enhancing the synergy with cisplatin (Wei et al. 2021).

Other approaches directly target the ERCC1-XPF complex, required for the incision step of NER. NSC 130,813 was identified as an inhibitor of ERCC1-XPF dimerization and was shown to sensitize cancer cells to cisplatin and mitomycin C in preclinical models (Jordheim et al. 2013). This finding supports the concept that inhibition of ERCC1-XPF may enhance the efficacy of DNA-damaging agents (Jordheim et al. 2013). Another preclinical strategy involves inhibition of the interaction between XPA and damaged DNA. Small molecules such as X80 were reported to inhibit XPA binding to DNA, suggesting that damage verification by XPA can also be pharmacologically targeted (Neher et al. 2010).

UCN-01 (7-hydroxystaurosporine), a cell cycle checkpoint inhibitor in Phase I clinical trials, was found to inhibit NER by disrupting the XPA-ERCC1 interaction and altering the phosphorylation of repair proteins (Jiang and Yang 1999). These findings suggest that combining NER inhibitors with platinum chemotherapy may represent a promising therapeutic strategy, particularly in patients with platinum-resistant tumors. However, most direct NER inhibitors remain at the preclinical stage, and further optimization is needed to improve potency, selectivity, pharmacokinetic properties, and safety (Neher et al. 2010; Jordheim et al. 2013; Gentile et al. 2020).

### NER and synthetic lethality

The success of PARP inhibitors in HRD tumors has inspired the search for analogous synthetic lethality strategies in NER. NER deficiency (dNER) can sensitize cancer cells to specific drug classes. An example is irofulven (an illudin S analog), an experimental drug that shows selective toxicity toward cells with TC-NER deficiency. Studies have demonstrated that an identifiable subset of clear cell renal cell carcinoma (ccRCC) tumors exhibits NER deficiency and is sensitive to irofulven, opening possibilities for targeted therapy (Prosz et al. 2023). Recently, a gene expression signature capable of predicting dNER in both cell lines and patient samples was developed (Wei et al. 2021). Importantly, none of the identified NER-deficient cell lines harbored mutations in any NER genes, suggesting that the prevalence of NER defects in cancer may be underestimated. Using this signature, multiple novel drugs exhibiting synthetic lethality with NER deficiency were identified and validated (Wei et al. 2021).

### NER and radiotherapy

Ionizing radiation induces DNA double-strand breaks, which are primarily repaired by NHEJ and HR pathways (Brook 2021). Radiation also generates oxidative and helix-distorting DNA lesions that interfere with transcription and replication and engage NER-dependent repair processes. Some oxidatively induced cyclopurine lesions (cyclo-dA/cyclo-dG) are strong transcription blockers and require TC-NER CSA/CSB and XPA for effective repair, where GG-NER is insufficient (Kaźmierczak-Barańska et al. 2021). At the phenotype level, in colorectal cancer, in vitro and in vivo models showed that *ERCC1* overexpression significantly increased radioresistance, suggesting it as a potential biomarker candidate (Zhang et al. 2018). Also, the *XPC* gene SNP rs2228001 mutation influences quality of life after radiotherapy; it causes more adverse effects during early and late recovery (Maćkowiak et al. 2024).

### NER and immunotherapy

Defects in NER lead to the accumulation of somatic mutations, increasing tumor mutational burden (TMB) and potentially generating neoantigens recognizable by the immune system. In silico analysis has associated mutations in ERCC-family genes with increased tumor immunogenicity and improved responses to immune checkpoint inhibitors, resulting in enhanced progression-free and overall survival. The mutant subgroup had a higher tumor mutation burden than the wild-type subgroup. Data suggest that NER gene alterations can benefit immune checkpoint inhibitor therapies, although the effect may be context- and tumor-type-dependent (Manguinhas et al. 2024b). *ERCC1* exon 3 skipping links DNA damage repair defects to cGAS-STING pathway activation in platinum-sensitive lung adenocarcinoma. The influence of cisplatin resistance suggests that *ERCC1* splicing is a potential biomarker for immunotherapy (Li et al. 2025). XPC group gene expression is correlated with anti-PD1 response in melanoma. Higher *ERCC3* expression was associated with better survival (Fischer et al. 2022).

### Role of transcription factors in NER regulation

A recent comprehensive review from 2024 highlights the complex interplay between transcription factors (TFs) and the NER pathway in cancer (Guneri-Sozeri and Adebali 2025). Factors such as TP53, c-Fos, AP-1, CLOCK-BMAL1, Sp1, and HIF1α regulate the expression of NER genes and influence DNA adduct formation and repair activity at their DNA binding sites. Modulating NER by targeting these transcription factors may represent an additional strategy to enhance therapeutic efficacy (Guneri-Sozeri and Adebali 2025).

## NER in in vitro studies across different cancer types

**Fig. 3:**
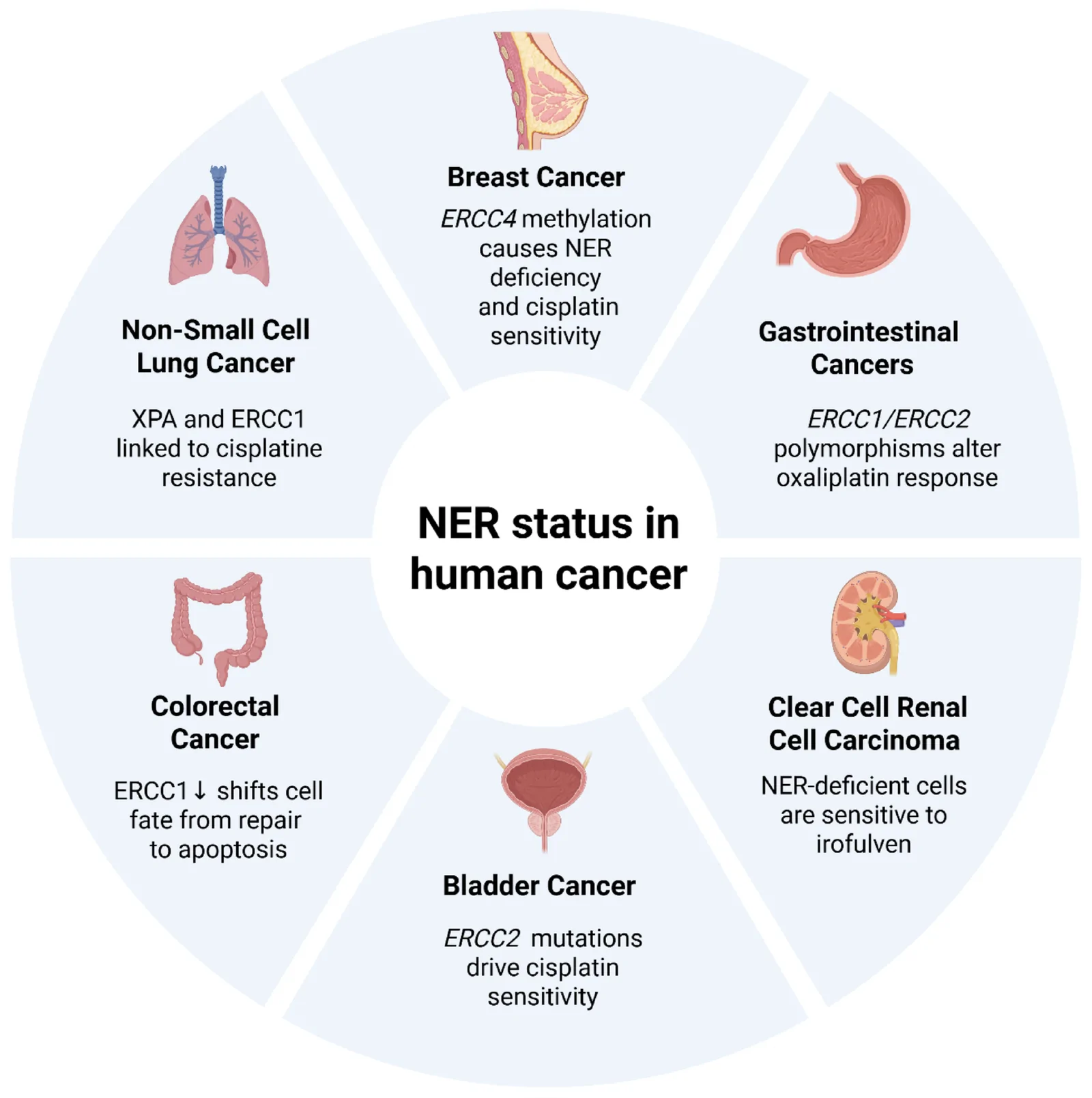
Role of nucleotide excision repair (NER) gene alterations in therapy response across different cancer types. Schematic overview illustrating the clinical relevance of alterations in key nucleotide excision repair (NER) pathway genes, particularly *ERCC1*, *ERCC2*, and *XPA*, across multiple human cancers. In breast cancer, *ERCC4* methylation is associated with NER deficiency and increased sensitivity to cisplatin. In gastrointestinal cancers, polymorphisms in *ERCC1* and *ERCC2* influence response to oxaliplatin-based chemotherapy. In clear cell renal cell carcinoma, NER-deficient cells demonstrate sensitivity to irofulven. Mutations in *ERCC2* are linked to cisplatin sensitivity in bladder cancer. Reduced *ERCC1* expression in colorectal cancer shifts cellular response from DNA repair toward apoptosis. In non-small cell lung cancer, alterations in *XPA* and *ERCC1* are associated with cisplatin resistance.

 In vitro cancer studies indicate that alterations in NER components may influence cellular response to DNA-damaging anticancer agents. The biological and clinical significance of these alterations varies between tumor entities. Figure 3 presents important clinical findings in the NER research area.

### Bladder cancer

Bladder cancer (BlC) represents one of the most advanced clinical and translational models for studying NER in oncology due to the frequent appearance of mutations. Somatic mutations in the *ERCC2* gene (encoding the XPD helicase) occur in approximately 10% of bladder tumors and are associated with increased cisplatin sensitivity (Van Allen et al. 2014; Börcsök et al. 2025a). Functional studies using a fluorescence microscopy-based NER assay demonstrated that the majority of ERCC2 helicase domain mutations are unable to support NER function (Li et al. 2019). Introduction of an *ERCC2* mutation into a bladder cancer cell line abrogated NER activity and was sufficient to drive cisplatin sensitivity in an orthotopic xenograft model (Li et al. 2019).

The Borcsok et al. study used the CRISPR-Select assay to quantitatively profile the functional effects of *ERCC2* mutations (Börcsök et al. 2025b). A single-allele editing version of this assay was also developed, enabling the assessment of heterozygous variants in their native context. Results confirmed that heterozygous helicase domain mutations markedly increase cisplatin sensitivity (Börcsök et al. 2025b). Importantly, comparisons with computational prediction algorithms revealed substantial discrepancies, underscoring the critical role of precision functional assays in interpreting mutation effects in clinically relevant contexts.

### Colorectal cancer

Studies on colorectal cancer (CRC) cell lines have provided important insights into the role of NER in drug-induced apoptosis. ERCC1 protein abundance serves as an indicator of the cellular decision between DNA repair and apoptosis (Erdemir Sayan et al. 2024). In SW480 and DLD1 cell lines treated with oxaliplatin, ERCC1 protein decreases and precedes mitochondrial depolarization and, in consequence, proteasomal degradation rather than caspase activity. Stabilization of ERCC1 using proteasome inhibitors attenuated the pro-apoptotic activity of oxaliplatin (Erdemir Sayan et al. 2024). Pharmacological inhibition of the XPF-ERCC1 complex enhanced the cytotoxicity of oxaliplatin chemo- and chemoradiotherapy treatment in colorectal cancer cell lines and in vivo models (Huang et al. 2023). These findings explain why clinical trials combining proteasome inhibitors with platinum derivatives have shown limited efficacy in treating carcinoma. In colorectal cancer cell lines, KRAS-mutant models were significantly more sensitive to oxaliplatin than KRAS wild-type cells. Although base *ERCC1* expression did not differ between groups, only KRAS wild-type cells were able to induce ERCC1 after oxaliplatin exposure. Importantly, KRAS silencing reduced oxaliplatin sensitivity and restored the capacity to induce ERCC1, whereas ERCC1 silencing increased oxaliplatin sensitivity and re-sensitized resistant cells. These findings suggest that the increased oxaliplatin sensitivity of KRAS-mutant colorectal cancer cells may result from their impaired ability to upregulate ERCC1 in response to treatment (Orlandi et al. 2015; Huang et al. 2023).

### Breast cancer

NER activity in breast cancer is still poorly characterized. A novel immunofluorescence-based cellular NER assay demonstrated that the majority of breast cancer cell lines are NER-proficient; however, a cell line with profound NER deficiency was identified (Rajkumar-Calkins et al. 2019). This deficiency was driven by epigenetic silencing of the *ERCC4* gene (encoding the NER nuclease XPF) through promoter methylation. *ERCC4* methylation also strongly correlated with mRNA and XPF protein expression in primary breast tumors. Re-expression of XPF in the *ERCC4*-deficient line rescued NER proficiency and cisplatin sensitivity but did not affect PARP inhibitor sensitivity (Rajkumar-Calkins et al. 2019). High *ERCC1* expression in metastatic triple-negative breast cancer treated with platinum-based chemotherapy was associated with poorer response, shorter progression-free and overall survival (El Baiomy and El Kashef 2017). These results indicate the potential of NER deficiency as a platinum sensitivity biomarker in breast cancer.

### Clear cell renal cell carcinoma

Clear cell renal cell carcinoma (ccRCC) has not benefited from synthetic lethality-based therapies due to a perceived lack of identified DNA repair deficiencies. However, a subset of ccRCC exhibits NER deficiency (Prosz et al. 2023). A functional NER assay based on monitoring the repair of UV-induced 6-4PP photoproducts enabled detection of varying degrees of NER deficiency across kidney cancer cell lines. Cells with high sensitivity to irofulven (A498, RXF393) displayed reduced NER activity, while resistant cell lines (786O, 769P) were proficient (Prosz et al. 2023). Whole exome sequencing data confirmed the presence of an NER-deficiency-associated mutational signature in a subset of clinical ccRCC biopsies (Prosz et al. 2023).

### Non-small cell lung cancer

In non-small cell lung cancer (NSCLC), expression of NER genes has long been investigated as a potential biomarker of response to platinum-based chemotherapy. Regulatory polymorphisms in NER genes (*ERCC1*, *ERCC2*, *XPA*, *XPC*) influence gene expression and correlate with survival outcomes in patients treated with platinum-based regimens (Zhang et al. 2017). In vitro studies on NSCLC cell lines confirm that XPA and ERCC1 protein levels correlate with cisplatin resistance (McGurk et al. 2006). Also, XPG inhibitors are potential compounds for enhancing cisplatin therapy in NSCLC, as shown by in silico and cell-based studies (Manguinhas et al. 2024b).

### Gastrointestinal cancers

In gastric and colorectal cancers treated with oxaliplatin-based regimens, NER gene polymorphisms – particularly *ERCC1* rs11615 and *ERCC2* rs13181 – are associated with therapeutic response, PFS, and OS, with significant ethnic differences (Yin et al. 2011). In hepatocellular carcinoma (HCC), frequent overexpression of NER genes, particularly *ERCC1*, *XPC*, and *XPA*, has been demonstrated, especially in tumors arising in cirrhotic livers (Fautrel et al. 2005). These findings suggest that NER pathway upregulation may contribute to the intrinsic chemoresistance commonly observed in HCC.

## NER in patient-based studies

### ERCC1 as a prognostic and predictive biomarker

*ERCC1* expression is one of the most intensively studied biomarkers of platinum therapy response in oncology. Meta-analyses encompassing thousands of patients with colorectal cancer, gastric cancer, and NSCLC have shown that the *ERCC1* rs11615 polymorphism (C > T) has predictive value for response to oxaliplatin-based chemotherapy, with significant differences between Asian and Caucasian populations (Yin et al. 2011; Olaussen and Postel-Vinay 2016). In Asian patients, the T allele (dominant allele) was associated with reduced response and shorter PFS and OS, while in Caucasians, an analogous role was played by the *ERCC2* rs13181 polymorphism (T > G) (Yin et al. 2011).

In lung cancer, immunohistochemical assessment of *ERCC1* expression in tumor tissue has been proposed as a marker for stratifying patients for platinum chemotherapy. However, results have been inconsistent, partly due to problems with antibody specificity for *ERCC1* detection and tumor heterogeneity (Simon et al. 2005; Olaussen and Postel-Vinay 2016). Despite these limitations, *ERCC1* remains one of the most promising NER biomarker candidates, and ongoing efforts focus on developing more reliable detection methods.

### ERCC2 as a biomarker in bladder cancer

Somatic mutations in the *ERCC2* gene represent the most clinically advanced NER biomarker. *ERCC2* mutations were significantly enriched in responders to neoadjuvant platinum-based chemotherapy in muscle-invasive bladder cancer (MIBC) (Van Allen et al. 2014). This finding was validated in an independent cohort, where 40% of responders vs. 7% of non-responders harbored an *ERCC2* mutation (*p* = 0.010, OR = 8.3) (Liu et al. 2016). In both cohorts, patients with *ERCC2* mutations had significantly longer overall survival (Van Allen et al. 2014; Liu et al. 2016).

A correlative analysis of the SWOG S1314 trial confirmed the association between DDR gene alterations (including *ERCC2*) and response to neoadjuvant cisplatin-based chemotherapy in MIBC patients (Iyer et al. 2024). In this study, 179 patients underwent next-generation sequencing (MSK-IMPACT), and results were correlated with pathologic response and survival. The study provided further evidence supporting the utility of *ERCC2* alterations as a biomarker of therapy response (Iyer et al. 2024).

### DDR gene panel as a composite biomarker

Single biomarkers, such as *ERCC2*, have limited sensitivity because not all platinum-sensitive tumors harbor mutations in this gene. Therefore, DDR gene panels encompassing *ATM*, *RB1*, *FANCC*, and *ERCC2* have been proposed as composite biomarkers (Plimack et al. 2024). Analysis of samples from the S1314 trial demonstrated that a mutation in any of these four genes predicted pathologic complete response (pT0) with an OR of 5.36 (95% CI: 2.05–14.02; *p* = 0.0006) (Plimack et al. 2024). The panel approach significantly increases detection sensitivity compared to single-gene analysis.

### NER-associated mutational signatures

NER deficiency leaves a characteristic footprint in the tumor genome, as a specific mutational signature. The SBS5 signature, associated with *ERCC2* mutations, has been identified in bladder cancer (D’Incalci and Galmarini 2010). Whole-genome sequencing analysis revealed that tumors serve as a diagnostic tool for identifying NER deficiency even in cases where direct analysis of NER gene mutations is uninformative, thereby expanding the pool of patients who might benefit from NER-directed therapies.

### *XPC* polymorphisms in head and neck cancer

Lopes-Aguiar et al. analyzed NER pathway polymorphisms in patients with HNSCC treated with cisplatin-based chemoradiotherapy. SNP variants in *XPC* and *XPD* genes were associated with treatment-related toxicity, and the *XPD* c934G > A variant was more frequent in patients with complete response (Lopes-Aguiar et al. 2017). Farnebo et al. analyzed DNA repair genes’ (*XPC*,* XPD*,* XRCC1*, XRCC3) SNP polymorphisms. In their study, XPC polymorphism A499V was associated with increased risk of laryngeal carcinoma (Farnebo et al. 2015). Our previous study analyzed the impact of the *XPC* gene polymorphism rs2228001 on radiotherapy response in patients with head and neck cancer (Maćkowiak et al. 2024). The results indicate that *XPC* genetic variants may modify sensitivity to ionizing radiation, expanding the range of potential applications for NER biomarkers beyond platinum-based chemotherapy. These findings suggest that NER pathway components may influence response to multiple DNA-damaging therapeutic modalities.

### NER in testicular germ cell tumors

Analysis of *ERCC1* and *XPA* expression in non-seminomatous TGCTs from patients in Mexico and Peru demonstrated that higher *ERCC1* expression is associated with cisplatin resistance, and that cisplatin treatment further increases expression of these proteins (Mendoza et al. 2013). Immunohistochemical assessment of ERCC1 protein in tumor sections confirmed its association with treatment outcomes. These clinical observations corroborate NER’s central role in determining platinum sensitivity and suggest that NER protein expression levels may serve as dynamic biomarkers that change during therapy. A study by Cierna et al. analyzed a large TGCT cohort and paired cell-line models and showed that high *XPA* expression was associated with worse overall survival and cisplatin-resistant phenotypes (Cierna et al. 2020).

## NER in clinical trials

### The SWOG S1314 trial—DDR biomarker validation in MIBC

The SWOG S1314 trial (NCT02177695) is a randomized Phase II study that evaluated two neoadjuvant chemotherapy regimens (gemcitabine + cisplatin vs. dose-dense MVAC) in patients with muscle-invasive bladder cancer (Iyer et al. 2024; Plimack et al. 2024). The correlative analysis encompassing 179 patients whose tumors underwent NGS sequencing (MSK-IMPACT) represents, to date, the broadest prospective validation of DDR biomarkers in this clinical context (Plimack et al. 2024).

Results confirmed that DDR gene mutations (including *ERCC2*) correlate with pathologic response to neoadjuvant chemotherapy. In particular, the four-gene panel (*ATM*, *RB1*, *FANCC*, *ERCC2*) demonstrated a strong association with pathologic complete response (pT0) (Iyer et al. 2024). These results form the foundation for designing future trials in which DDR gene status will determine treatment strategy.

### Bladder-sparing trials—NCT03609216 and RETAIN

Ongoing clinical trials aim to validate DDR mutations (including *ERCC2*) as biomarkers enabling a bladder-sparing approach. The hypothesis holds that patients with *ERCC2* mutations who achieve pathologic complete response to chemotherapy may avoid radical cystectomy (Galsky et al. 2023). The RETAIN trial (Phase II) evaluates an adaptive approach in which the decision about surgery is based on chemotherapy response and the tumor’s genomic profile (Geynisman et al. 2025). If validated, this approach could improve the treatment paradigm for MIBC by preserving quality of life while maintaining oncological outcomes.

### ERCC1 as prognostic biomarker in HNSCC

In a planned substudy of a randomized phase II trial (NCT00410826) in patients with locally advanced HNSCC, elevated ERCC1-XPF protein expression was associated with shorter progression-free survival after chemo-radiotherapy. The specific FL297 and 4F9 antibodies were used to observe this effect and remained prognostically relevant in HPV-associated disease, supporting ERCC1-XPF as an adverse outcome biomarker independent of HPV status (Bauman et al. 2013).

### Trabectedin in clinical trials

Trabectedin is approved for the treatment of advanced soft tissue sarcomas and recurrent ovarian cancer (in combination with pegylated liposomal doxorubicin). Its unique transcription-coupled nucleotide excision repair (TC-NER)-dependent mechanism of action makes it particularly interesting in the context of precision medicine (D’Incalci and Galmarini 2010; Son et al. 2024). Trabectedin binds to DNA and induces lesions that are preferentially processed by the TC-NER pathway, leading to cytotoxicity specifically in cells with active transcription-coupled repair. In this context, the TRABI-Seq method, developed in 2024, enables mapping genome-wide TC-NER activity by sequencing DNA regions affected by trabectedin-induced damage and repair (Son et al. 2024). The approach leverages the fact that trabectedin generates DNA adducts whose processing depends on TC-NER, allowing the identification of genomic loci where this pathway is active. Using this strategy, the study demonstrated that patterns of trabectedin-induced damage and repair can serve as a proxy for TC-NER activity across the genome. These findings suggest that trabectedin may have a dual role: not only as a therapeutic agent exploiting TC-NER for selective cytotoxicity, but also as a molecular probe to functionally assess TC-NER activity in tumors. Such an approach could support patient stratification by identifying tumors with high TC-NER activity, which are more likely to respond to trabectedin-based therapy.

### NER inhibitors—clinical prospects

Although direct NER inhibitors have not yet entered routine clinical trials, progress in identifying therapeutic targets has been significant. The current preclinical landscape includes compounds targeting XPA-ERCC1 interaction, ERCC1-XPF dimerization, ERCC1-XPF nuclease function, and XPA-DNA binding (Neher et al. 2010; Jordheim et al. 2013; Gentile et al. 2016, 2020). The XPA-ERCC1 interaction remains the primary pharmacological target, and modern in silico drug design methods have enabled identification of inhibitor candidates with improved efficacy (Gentile et al. 2016, 2020). Inhibition of this interaction may reduce NER efficiency and increase the persistence of platinum-induced DNA lesions (Gentile et al. 2016, 2020). The ERCC1-XPF complex is also an important target because it performs the 5′ incision during NER and contributes to repair of platinum-induced DNA damage (Jordheim et al. 2013). Compound NSC 130,813 [4-[(6-chloro-2-methoxy-9-acridinyl)amino]-2-[(4-methyl-1-piperazinyl)methyl]] is a representative ERCC1-XPF dimerization inhibitor that has shown sensitizing activity in combination with cisplatin and mitomycin C in preclinical models (Jordheim et al. 2013). Inhibitors of XPA-DNA binding, including X80, provide additional proof of concept that early steps of NER can be pharmacologically inhibited (Neher et al. 2010). In parallel, strategies based on synthetic lethality in NER-deficient tumors (e.g., irofulven in ccRCC) are at the preclinical and early clinical trial stages (Prosz et al. 2023).

Despite these promising findings, clinical translation remains limited. The main challenges include insufficient selectivity, limited in vivo validation, potential toxicity in normal tissues, and the need for predictive biomarkers (Neher et al. 2010; Jordheim et al. 2013; Gentile et al. 2020). Future studies should determine whether ERCC1, XPF, XPA expression, or functional NER activity can identify patients most likely to benefit from NER inhibition (Gentile et al. 2020; Prosz et al. 2023).

An emerging approach is combining NER modulation with immunotherapy. NER-deficient tumors, characterized by higher TMB, may particularly benefit from the combination of platinum drugs with immune checkpoint inhibitors. a Phase II trial evaluating gemcitabine, cisplatin, and nivolumab as an organ-sparing treatment in MIBC (2023) exemplifies this research direction (Teo et al. 2018; Galsky et al. 2023). Results demonstrated encouraging rates of pathologic complete response, supporting the continued investigation of combined chemo-immunotherapy approaches guided by DNA repair biomarkers. Pharmacological NER inhibition may also increase DNA damage burden and tumor immunogenicity, providing a rationale for future combinations with immune checkpoint blockade (Teo et al. 2018; Galsky et al. 2023). However, this concept remains experimental and requires further preclinical validation.

## Conclusions and future perspectives

Nucleotide excision repair (NER) plays a central role in cancer biology, influencing both carcinogenesis and therapeutic response. This review highlights the rapid progress of knowledge in this field. The most significant achievements of recent years include:

1. Identification of *ERCC2* mutations as a predictive biomarker of cisplatin response in bladder cancer, validated across multiple independent cohorts and clinical trials, including the SWOG S1314 study.
2. Elucidation of trabectedin’s mechanism of action as dependent on abortive TC-NER, together with the development of TRABI-Seq, a method enabling genome-wide mapping of TC-NER activity.
3. Discovery of the role of *ERCC1* as an indicator of the repair-versus-apoptosis decision and explanation of why proteasome inhibitors attenuate platinum efficacy.
4. Development of a gene expression signature for predicting NER deficiency and identification of novel synthetic lethality strategies.

Future perspectives include prospective validation of NER biomarkers in randomized clinical trials, development of clinically applicable NER inhibitors, integration of NER status with immunotherapy decision-making, and expansion of applications beyond bladder cancer to other platinum-treated malignancies. a deeper understanding of epigenetic regulation of NER genes and the role of transcription factors in modulating pathway activity will also be crucial. As the field moves toward clinical implementation, the convergence of functional genomics, computational biology, and clinical trial design promises to transform NER-directed precision oncology from a research concept into a therapeutic reality.

## Data Availability

To prepare Figs. 1, 2 and 3, we used existing data from publications cited in the manuscript. The data is available upon request.

## Declarations

### Usage of Artificial Intelligence

The authors confirm that artificial intelligence tools were not used in the preparation or analysis of this manuscript.
